# Hydrothermal-derived carbon as a stabilizing matrix for improved cycling performance of silicon-based anodes for lithium-ion full cells

**DOI:** 10.3762/bjnano.9.223

**Published:** 2018-09-05

**Authors:** Mirco Ruttert, Florian Holtstiege, Jessica Hüsker, Markus Börner, Martin Winter, Tobias Placke

**Affiliations:** 1 University of Münster, MEET Battery Research Center, Institute of Physical Chemistry, Corrensstraße 46, 48149 Münster, Germany; 2 Helmholtz Institute Münster, IEK-12, Forschungszentrum Jülich GmbH, Corrensstraße 46, 48149 Münster, Germany

**Keywords:** LIB full cell, lithium-ion batteries, prelithiation, silicon/carbon composite, solid–electrolyte interphase (SEI)

## Abstract

In this work, silicon/carbon composites are synthesized by forming an amorphous carbon matrix around silicon nanoparticles (Si-NPs) in a hydrothermal process. The intention of this material design is to combine the beneficial properties of carbon and Si, i.e., an improved specific/volumetric capacity and capacity retention compared to the single materials when applied as a negative electrode in lithium-ion batteries (LIBs). This work focuses on the influence of the Si content (up to 20 wt %) on the electrochemical performance, on the morphology and structure of the composite materials, as well as the resilience of the hydrothermal carbon against the volumetric changes of Si, in order to examine the opportunities and limitations of the applied matrix approach. Compared to a physical mixture of Si-NPs and the pure carbon matrix, the synthesized composites show a strong improvement in long-term cycling performance (capacity retention after 103 cycles: ≈55% (20 wt % Si composite) and ≈75% (10 wt % Si composite)), indicating that a homogeneous embedding of Si into the amorphous carbon matrix has a highly beneficial effect. The most promising Si/C composite is also studied in a LIB full cell vs a NMC-111 cathode; such a configuration is very seldom reported in the literature. More specifically, the influence of electrochemical prelithiation on the cycling performance in this full cell set-up is studied and compared to non-prelithiated full cells. While prelithiation is able to remarkably enhance the initial capacity of the full cell by ≈18 mAh g^−1^, this effect diminishes with continued cycling and only a slightly enhanced capacity of ≈5 mAh g^−1^ is maintained after 150 cycles.

## Introduction

Since their market launch in 1991, the energy density of lithium-ion batteries (LIBs) has increased steadily. However, further improvements in terms of power density and energy density are essential to meet the rising requirements for automotive applications, e.g., extended driving range and fast charging ability. Such improvements can either be achieved by the optimized engineering of cell components or the development of new cell chemistries with advanced active materials [1–6].

In this context, it is remarkable that the LIB cell chemistry concerning the negative electrode (anode) of commercial cells is still quite similar to that of the very first LIBs, based on carbonaceous anode materials. There are several good reasons why carbonaceous anode materials, especially graphite, are still state of the art. For example, they maintain a high specific capacity (372 mAh g^−1^) compared to cathode materials, high electrochemical stability in suitable electrolytes, a low operation potential (0.2 V vs Li/Li^+^), low voltage hysteresis, low cost, and are environmentally friendly [7–8]. Nonetheless, alternative anode materials, such as silicon (Si) and tin (Sn), have aroused great interest in the last decade with the aim to replace graphite, as these materials offer considerably higher theoretical, specific capacities of 3,579 mAh g^−1^ and 990 mAh g^−1^, respectively, compared to that of graphite [9–12]. The high capacity of Si results from a different lithium-ion storage mechanism compared to graphite: while graphite intercalates Li-ions into its host structure, Si “alloys” with Li (or more precisely, forms various intermetallic phases) at a maximum stoichiometry of Li_15_Si_4_ at ≈50 mV vs Li/Li^+^ [13]. Si is considered as the most promising candidate to replace graphite because, aside from the high gravimetric and volumetric capacity, this material can be obtained from inexpensive and highly available precursors (e.g., silicon dioxide) and still offers a relatively low operating potential (≈0.4 vs Li/Li^+^). Therefore, high cell voltages can be achieved using appropriate cathode materials [10,12,14].

Based on energy density calculations, it was reported that the total specific capacity can significantly be increased on the cell level by the application of high capacity anode materials. Considering the specific capacities of cathode materials that are available today (≤200 mAh g^−1^), these calculations show that it is reasonable to aim for anode materials with specific target capacities of ≈1000–1200 mAh g^−1^, as a further increase to even higher capacities would yield only a small additional energy gain [1,15]. In some commercial cells, Si is already added in small amounts (≤5 wt %) to the graphite anode [5].

Yet, there are several major drawbacks that have to be overcome for a successful application of Si-based anodes, i.e., the low electronic conductivity, as well as the huge volume changes of ≈300–400% during the lithiation/delithiation process [15–17]. The latter issue leads to severe mechanical stress and causes rupturing of the electrode, electronic contact loss between active material and current collector/conductive carbon network and pulverization. Furthermore, the drastic volume changes during cycling hinder the formation of a dimensionally stable solid electrolyte interphase (SEI), as it is known for carbonaceous anodes, formed on the negative electrode surface from electrolyte decomposition products in the first charge/discharge cycles [18–20]. In the case of Si anodes, the SEI formation is an ongoing process because of the recurring breakage of the already formed SEI and exposure of fresh Si to the electrolyte. Consequently, a very thick SEI may form after several cycles, affecting the reaction kinetics detrimentally. All these aforementioned factors contribute to a decreasing capacity with each cycle, either due to consumption of active Li, trapping of Li in disconnected Si or a growing resistivity [17,21–25].

With the aim to tackle these problems and to obtain Si anodes with a stable cycling performance at high capacity, several promising approaches have been reported in the recent years. Some of the concepts that led to enormous improvements include the adaption of well-know concepts from Sn-based materials [11,26], such as reduction of the Si particle size to the nanoscale [15,27–29], the improvement of binders for composite electrodes [30–33], the search for effective SEI-forming electrolyte additives [34–35], as well as the embedding of Si into different matrix materials [36–41]. The general idea behind the latter concept is the combination of Si with a second phase, which can be either active or inactive towards lithiation itself. This phase should be able to provide high mechanical stability and accommodate the volumetric changes of Si, alleviating the aforementioned detrimental effects. Thus, these matrices should exhibit no (or less) volume changes compared to Si, and ideally, offer high electronic conductivity. Besides carbon-based matrices, intermetallic, silicide phases consisting of Si and different metals, such as Mg [42–43], Fe [40], Cr [44] or Ni [39,45] are the most prominent representatives of this approach. There is a vast amount of publications focusing on carbon/silicon composites (Si/C), dealing with the incorporation of Si into a variety of different carbon materials, such as graphite, graphene sheets [46–47], porous carbon structures [37–38,48] or the coating of Si using different precursors as carbon sources [49–51]. One simple method to form amorphous carbon structures, depicts the hydrothermal synthesis of carbohydrates [52]. Due to the fact, that this synthesis can be carried out at mild reaction conditions (<200 °C), using water as a solvent and carbohydrates as a carbon source, this process is environmentally friendly and quite inexpensive. Cakan et al. [41] showed that Si nanoparticles (Si-NPs) can be embedded in spherical hydrothermal carbon via a simple one-step hydrothermal process and Hu et al. [53] used hydrothermal carbonization to form a thin carbon and SiO*_x_* layer around Si-NPs and reported a great improvement in cycling stability compared to pure Si-NPs. Shen et al. [37] also used a hydrothermal method to synthesize a pomegranate-inspired Si/C composite with Si-NPs distributed within a porous carbon structure and reported a capacity of 581 mAh g^−1^ after 100 cycles with a capacity retention of ≈77%. These previously mentioned publications clearly point out the potential of hydrothermal-derived carbons as promising matrix material, however, they do not investigate the influence of different Si contents on the resilience of the carbon matrix and possible limitations of this approach. Furthermore, the electrochemical characterizations in these publications do not include the application in a real LIB full cell set-up, but only investigations vs Li-metal counter electrodes (“half-cell” set-up).

In general, it should be stated that even though some impressive cycling results of Si-based anode materials with stable cycling performances at high capacities have been reported in the recent years, most of these results are obtained vs Li-metal electrodes. This means that the amount of Li in this cell set-up is unlimited and capacity fading related to active lithium loss cannot be detected. In a LIB full cell set-up, however, the amount of active Li is limited by the cathode material [25]. The restricted Li content is a very critical aspect regarding the application of Si in a full cell set-up, considering the lower Coulombic efficiencies (CEs) of Si-based anodes, especially in the first cycle. A powerful method to counterbalance the active Li loss in the first cycle and thus improve the energy density of the cell, is prelithiation, meaning that additional active Li is added to the system before the operation of the cell [54–55]. In this context, Chevrier et al. [56] developed an idealized model, correlating prelithiation with variations in energy density. Depending on the amount of added Li, prelithiation can compensate the irreversible capacity loss of the negative electrode in the first cycle and, therefore, improve the energy density. Alternatively, when further Li is added, prelithiation can also be used to create a Li reservoir in order to compensate for active lithium loss with ongoing cycling and increase the cycle life of a cell. Further, Marinaro et al. [57] reported an approach to add Li to Si anodes by depositing a suspension of stabilized Li-metal powder (SLMP) in toluene onto an electrode via airbrushing, leading to significantly improved first cycle CEs and enhanced cycle life of the prelithiated electrodes in comparison to the non-prelithiated electrodes.

In this work, we use a simple hydrothermal process, followed by a carbonization step to synthesize Si/C composites, in which Si-NPs are homogeneously dispersed within an amorphous carbon matrix. The aim of the applied synthesis route is to combine the beneficial properties of Si and carbon in a Si/C composite material with high specific capacity, good rate performance and long-term cycling stability. Thereby this contribution lays focus on the influence of the Si to C ratio to identify the chances and limitations of the applied hydrothermal carbon matrix approach. The synthesized materials are characterized regarding their composition, structure and morphology. The Si/C composites are also investigated in terms of their electrochemical performance, i.e., by rate performance and long-term cycling experiments. The most promising composite material is also characterized in a LIB full cell set-up to verify the applicability in a real cell system. Further, the influence of prelithiation on the LIB full cell long-term capacity retention is studied.

## Experimental

### Synthesis of silicon/carbon composites and of the pure hydrothermal carbon matrix

Silicon/carbon (Si/C) composites and the pure carbon matrix were synthesized in a hydrothermal process using a solution of anhydrous D-glucose (Fisher Scientific) in water (0.75 mol L^−1^) as the carbon source in the presence or absence of commercially available Si-NPs (100 nm, NANO Si, Creavis) with D-glucose:Si weight ratios of 77.5:1 and 45.5:1, respectively. Therefore, D-glucose was dissolved in deionized (DI) water by stirring for 15 min. At the same time, in the case of Si-containing samples, Si-NPs were dispersed in a small amount of DI water and added to the D-glucose solution. A specific volume was set to obtain a glucose concentration of 0.75 mol L^−1^. The (combined) solution was treated in an ultrasonic bath for 45 min in order to break agglomerates and obtain a homogeneous suspension. For the hydrothermal treatment, the solution was transferred into a pressure reactor (Parr Instrument Company), equipped with a 600 mL polytetrafluoroethylene (PTFE) liner and two six-blade impellers, one near the bottom and one near the surface of the solution. The hydrothermal treatment consisted of a 150 min heating-up phase to 180 °C, followed by a holding phase of 330 min at a nitrogen prepressure of 4.8 bar and a stirring rate of 200 rotations per minute (rpm). The product of the hydrothermal process was collected by filtration using a membrane with 0.2 µm pores (Merck Millipore) and washed with DI water, ethanol and acetone and dried at 60 °C in ambient atmosphere.

Afterwards, the dried products were carbonized at a temperature of 900 °C for six hours in an argon atmosphere, applying a heating ramp of 300 °C h^−1^, in order to remove heteroatoms and increase the electronic conductivity of the materials.

### Electrode preparation

Composite electrodes with a composition of 90 wt % active material (Si/C composite, pure carbon matrix or mixture of pure carbon matrix and Si-NPs), 5 wt % sodium carboxymethyl cellulose (Na-CMC) as binder (Walocel CRT 2000 PPA 12; Dow Wolff Cellulosics) and 5 wt % conductive agent (C-nergy Super C65; Imerys Graphite & Carbon) were prepared by coating a dispersion of the aforementioned materials and water onto a dendritic copper foil (Carl Schlenk AG). At first, the Na-CMC was dissolved in DI water, followed by the addition of the conductive agent and of the active material. After dispersing the electrode paste for one hour at 10,000 rpm (VMA-GETZMANN GmbH), it was cast onto a previously purified (ethanol) dendritic copper foil with a speed of 50 mm s^−1^ using a doctor blade technique (Zehntner GmbH) in combination with an automatic film applicator (Sheen Instruments). A wet coating thickness of 100 µm was applied, leading to an average mass loading of ≈2.0 mg cm^−2^. After drying for one hour at 50 °C, circular electrodes with a diameter of 12 mm were punched out and dried under reduced pressure (<0.05 mbar) at 170 °C for at least 24 h and stored in a glove box with argon atmosphere.

#### Cell assembly and electrochemical investigations

Electrochemical investigations were carried out in a three electrode configuration using Swagelok-type T-cells that were assembled in a glove box (UNIlab, MBraun) with argon atmosphere and H_2_O and O_2_-values below 0.1 ppm. The composite electrodes containing the synthesized materials were used as working electrodes (WE), while lithium metal (Li; Albemarle Corporation) was used as counter and reference (RE) electrodes. In the full cell set-up, the Si/C composite electrodes were cycled vs lithium nickel manganese cobalt oxide (LiNi_1/3_Mn_1/3_Co_1/3_O_2_, NMC-111; Umicore; D90: 17.0 µm; mass loading ≈6.5 mg cm^−2^) electrodes with an active material: polyvinylidene difluoride (PVDF) binder: Super C65 composition of 93:4:3 wt % and Li-metal was used as reference electrode. A six-layered polyolefin separator (Freudenberg 2190; diameter: 13 mm) soaked with 140 µL of electrolyte was placed between negative and positive electrode. The reference electrode was spaced apart from the other electrodes by a three-layered separator (diameter: 8 mm) containing 60 µL of electrolyte. The used electrolyte was a mixture of ethylene carbonate (EC) and dimethyl carbonate (DMC) in a ratio of 1:1 by weight, 1 M LiPF_6_ (LP30, BASF) plus 5 vol % of fluoroethylene carbonate (FEC, BASF).

A Maccor Series 4000 automated test system (Maccor) was used to carry out constant current charge (=lithiation)/discharge (=delithiation) experiments. The cut-off potentials during the long-term cycling experiments were set as 0.01 V vs Li/Li^+^ and 1.50 V vs Li/Li^+^. During the rate performance experiments, cut-off potentials of 0.02 V vs Li/Li^+^ and 1.50 V vs Li/Li^+^ were chosen in order to avoid Li-metal plating at high charging rates. In the rate performance studies, specific charge/discharge currents between 40 mA g^−1^ and 1,000 mA g^−1^ were applied. Long-term cycling experiments were carried out at a specific charge/discharge current of 400 mA g^−1^ after three formation cycles with a specific current of 80 mA g^−1^.

In the full cell set-up an anode/cathode capacity balancing (*Q*_A_/*Q*_C_) of 1:1 was used and the cells were cycled at a cell voltage of 3.0 V and 4.3 V. In addition, the reference electrode was used to monitor the anode potential. After three formation cycles with 10 mA g^−1^, a specific current of 100 mA g^−1^ was applied for cycling.

The currents refer to the active material mass of the working electrode in Li-metal cells or to the active material mass of the NMC-111 cathode in the full cell set-up, respectively.

Electrochemical prelithiation was carried out by performing one formation cycle in a Si/C vs Li-metal cell at a charge/discharge current of 50 mA g^−1^, followed by disassembling the cell in a glove box and assembling of a full cell using the prelithiated Si/C electrode as the negative electrode.

#### Characterization methods

A Bruker Senterra Raman microscope (Bruker Optics Inc.) was used to record the Raman spectra using a green laser with a wavelength of 532 nm and a laser power of 5.00 mW.

X-ray diffraction (XRD) patterns in a 2θ range of 20° to 80° were recorded with the help of a Bruker D8 Advance X-ray diffractometer (Bruker AXS GmbH) with a Cu Kα-wavelength of λ = 0.154 nm and a step size of 0.039°.

Thermogravimetric analysis (TGA) was carried out in a temperature range between 25 °C and 800 °C on a TGA Q500 (TA Instruments) in an oxygen/nitrogen atmosphere (nitrogen flow: 10 mL min^−1^, oxygen flow: 25 mL min^−1^) in order to determine the Si content. A heating ramp of 20 °C min^−1^ was applied.

Scanning electron microscopy (SEM) with a field emission gun (Schottky-type) was used to investigate the morphology of the synthesized composite materials. Cycled electrodes were analyzed after washing with DMC and drying in an argon filled glovebox. Multiple areas per sample were analyzed using an Auriga CrossBeam workstation from Zeiss at an acceleration voltage of 3 kV. Energy-dispersive X-ray spectroscopy (EDX) measurements were used to investigate the elemental composition of the composite materials using an acceleration voltage of 20 kV. The EDX signal was detected by an X-Max 80 mm^2^ detector and evaluated with the INCA software, both from Oxford Instruments. Cross-sections were prepared by a focused ion beam (FIB) milling process using gallium ions extracted from a high brightness liquid metal ion source.

Nitrogen adsorption experiments were performed on a 3Flex Physisorption device (Micromeritics GmbH) at the temperature of liquid nitrogen (−196 °C). Before the measurements, the samples were degassed at 200 °C for two days. The surface areas were calculated in accordance to the BET (Brunauer–Emmett–Teller) theory.

Tap densities were measured using an AUTOTAP tapped density analyzer (Quantachrome Instruments). Therefore, the samples were accurately weighed, filled in a measuring cylinder and tapped for 5000 times before the volume was determined.

## Results and Discussion

### Morphology and internal structure of the synthesized Si/C materials

**Figure 1:**
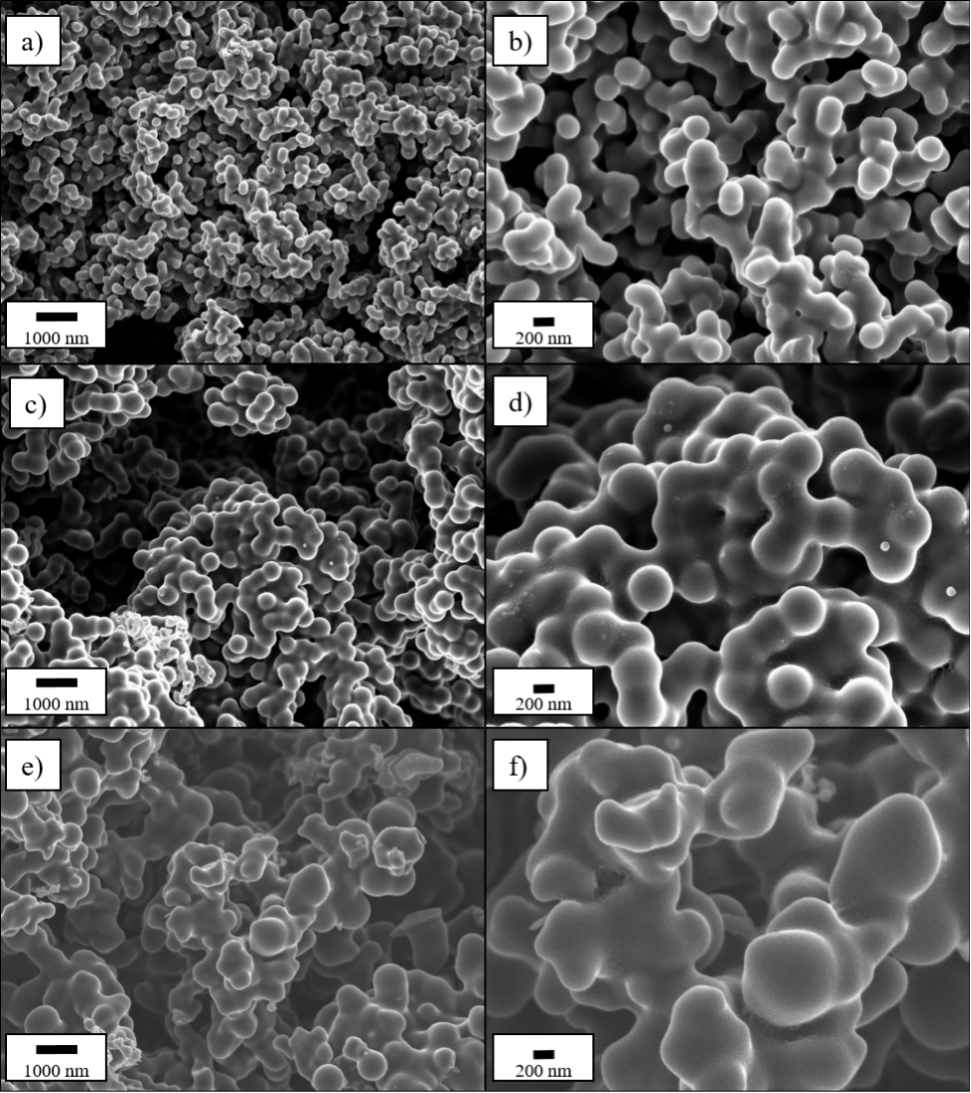
SEM micrographs of the different synthesized Si/C composite materials with a carbon to silicon ratio of 100:0 (a, b), 90:10 (c, d) and 80:20 (e, f) in a magnification of 10k× (a, c, e) and 25k× (b, d, f).

**Figure 2:**
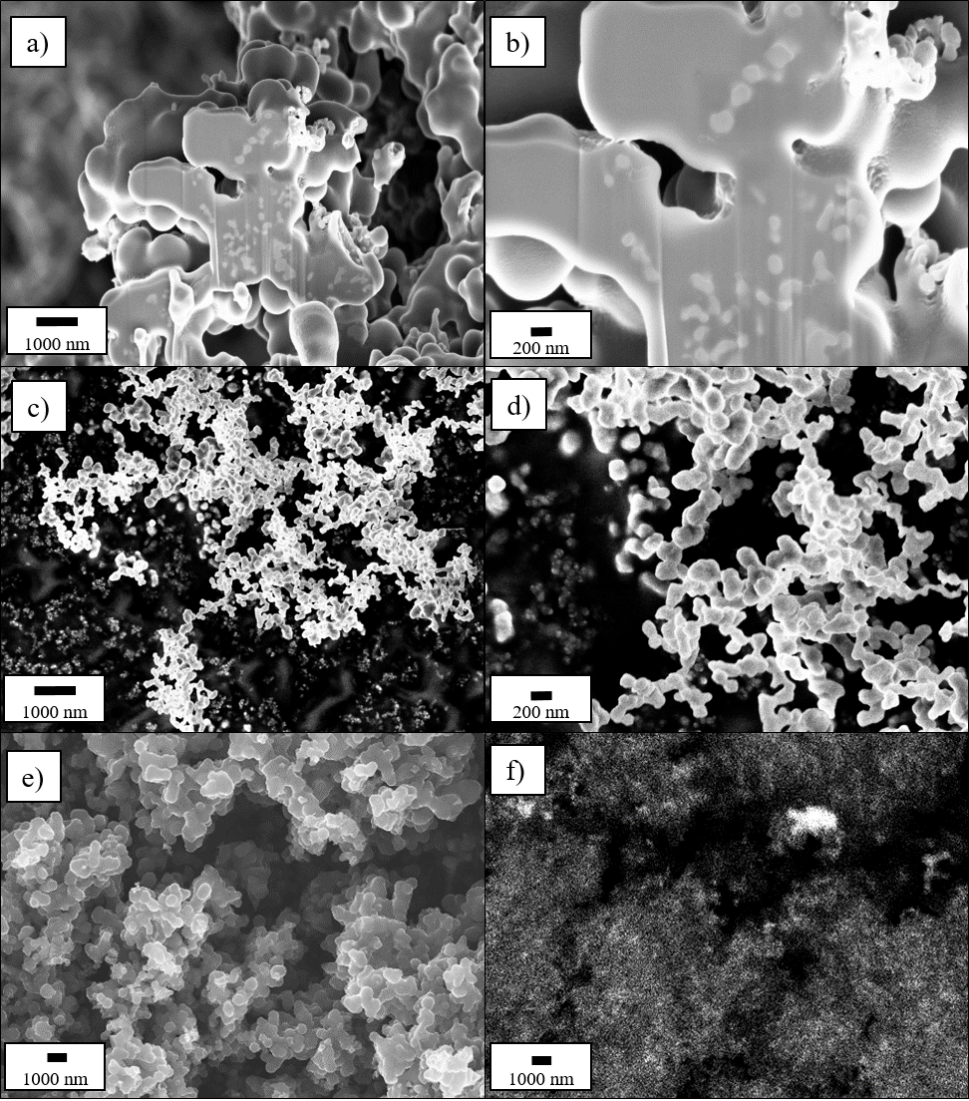
FIB-SEM cross section of the C:Si 80:20 composite (a, b) and SEM micrographs of the pure Si-NPs (c, d) and an EDX mapping of the C:Si 80:20 composite, showing the Si distribution (=white areas) within the matrix (f) and the corresponding SEM micrograph (e).

The stoichiometry of silicon nanoparticles (Si-NPs) during the hydrothermal process was calculated with the goal to obtain Si/C composites containing 0 wt % (pure carbon matrix), 10 wt % (C:Si 90:10) and 20 wt % (C:Si 80:20) of Si, homogeneously embedded in a carbonaceous matrix. The morphology of the synthesized samples was investigated by means of SEM as presented in Figure 1. From Figure 1a and Figure 2b it can be seen that the chosen reaction conditions lead to spherical carbon particles with a diameter of ≈200 nm that are quite strongly fused together and, therefore, form large agglomerates. Chain-like aggregates of spherical carbon particles, were also found by Tien et al. [58], when they synthesized carbon spheres by thermal decomposition. Jin et al. [59] and Kristianto et al. [60] also reported the presence of conglomerated carbon spheres rather than single spherical particles, which might be caused by extended reaction times or the cooling phase after the synthesis [61]. A continuous, interconnected network of nanospheres was also reported by Xia et al. [62] during the synthesis of carbon spheres containing electrocatalysts for oxygen reduction reactions. Heckmann et al. [63] investigated the use of high-temperature-treated hydrothermal carbon spheres as cathode materials for dual-ion cells and found spherical particles up to a heat treatment temperature of 2100 °C, while at temperature of 2400 °C, they observed the additional formation of rod-shaped particles.

The addition of Si-NPs results in a visible increase in primary particle size, which is especially stressed for the sample with the higher Si content of 20 wt % (Figure 1e and 1f). The spherical shape of the single particles is still recognizable for the C:Si 90:10 sample, despite the strong particle fusion (Figure 1c and 1d), whereas the morphology of the C:Si 80:20 sample is more irregular-shaped and not as uniform and round-shaped as for the C:Si 90:10 sample.

The SEM micrographs also show that nearly no Si-NPs are located outside of the matrix, indicating a successful embedding of Si into carbon. To further verify this assumption, the internal structure of the C:Si 80:20 sample was investigated with the help of FIB-SEM and EDX to obtain a cross-section of the material and identify the Si distribution inside the composite (Figure 2). The cross section in Figure 2a and 2b shows several lighter spots located inside the matrix material. In comparison with Figure 2c and 2d which show the pure Si-NPs that were added during the synthesis, the similarities in shape and size to the Si particles (=white spots) in Figure 2a and 2b can be seen. The EDX mapping results in Figure 2e and 2f also supports the results from the FIB-SEM investigations that Si is homogeneously distributed within the carbon matrix. For comparison reasons, SEM micrographs of a physical mixture of the pure carbon matrix and the pure Si-NPs in a weight ratio of 80:20, where the Si-NPs are not embedded in the carbon matrix, are presented in Figure S1 (Supporting Information File 1).

### Determination of the silicon content and structural characteristics

**Figure 3:**
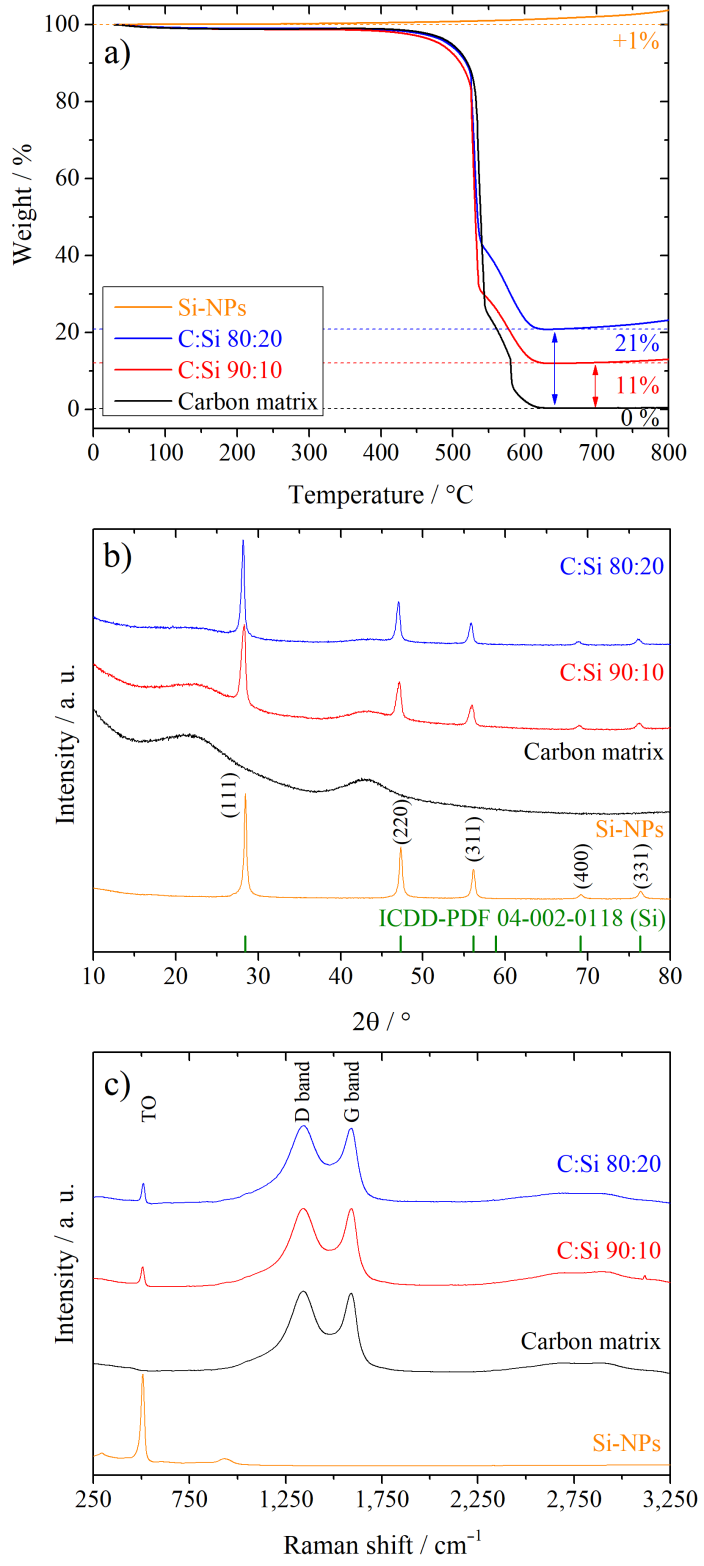
TGA results (a), XRD patterns (b) and Raman spectra (c) of the Si/C composites with a carbon to silicon ratio of 100:0, 90:10, 80:20 and the pure Si-NPs.

To identify the actual Si content of the Si/C composites, TGA was carried out in an oxidative atmosphere, as presented in Figure 3a. While the pure carbon matrix burns off completely and the remaining weight at a temperature of 630 °C is ≈0%, the Si-containing samples exhibit a small plateau at a temperature of ≈630 °C where the remaining weight is constant. Due to the fact that the pure Si-NPs show only an insignificant weight gain up to 650 °C of ≈1%, caused by the beginning oxidation of Si and the formation of silicon dioxide, the remaining weight of the plateau for the Si/C composites can be considered as the Si content of these materials [39]. The Si contents determined in this way amount to 11 wt % for the C:Si 90:10 composite and 21 wt % for the C:Si 80:20 composite, which is close to the desired values and means that the C:Si ratio can be controlled accurately by the Si to glucose ratio during the first step of the synthesis.

The XRD patterns of the Si/C composites, the pure carbon matrix and the pure Si-NPs are depicted in Figure 3b. Both Si/C composites exhibit sharp reflections that are characteristic for the diamond structure of crystalline silicon (ICDD-PDF 04-002-0118, space group 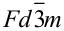 (no. 227)) at 2θ values of 28.4°, 47.3°, 56,1°, 69,1° and 76.3°, as can be seen in comparison to the pattern of the pure Si-NPs [39]. In contrast to the Si-containing materials, the pure carbon matrix exhibits no sharp reflections but two broad humps at 2θ values of ≈22° and ≈43° that are also observable for both Si/C composites. This indicates an amorphous structure of the carbon and can be explained with the carbonization temperature of 900 °C that is way below the temperature needed to grow large crystalline, graphitic domains [64–65]. This carbonization temperature was chosen with the aim to synthesize a material with a porous, amorphous structure that is able to accommodate the volumetric changes of the Si during the lithiation/delithiation process. The formation of silicon carbide (SiC) or any other crystalline SiO*_x_* phases in detectable amounts is also avoided at this temperature as can be reasoned from the absence of any further sharp reflections, other than that of the crystalline Si.

The amorphous nature of the carbon matrix was also confirmed with the help of Raman spectroscopy, as depicted in Figure 3c. Both Si/C composites, as well as the pure carbon matrix exhibit two bands at 1,345 cm^−1^ and 1,593 cm^−1^ that show a similar intensity and strong overlap. These bands can be attributed to the *D*- and *G*-band and are characteristic for amorphous or disordered carbons [41,64]. The band at ≈510 cm^−1^ originates from a transverse optical mode of Si [27,53].

In order to determine the achievable energy density of the synthesized Si/C composite materials, the tap density of these materials was determined and summarized in Table S1 (Supporting Information File 1). In general, nanometer-sized materials suffer from a low tap density, which is detrimental in terms of energy density (Wh L^−1^). In comparison to the pure Si-NPs (tap density of 0.13 g cm^−3^), the tap densities of the Si/C composites are considerably higher, however, they are still quite low compared to state-of-the-art micrometer-sized graphite anode materials (typically ≥1 g cm^−3^). Thus, further improvements are mandatory to achieve higher tap densities and, therefore, practical energy densities for mobile applications. While the pure carbon matrix exhibits a tap density of ≈0.16 g cm^−3^, the tap densities of the C:Si 90:10 and C:Si 80:20 sample increase to ≈0.19 g cm^−3^ and ≈0.24 g cm^−3^. For a meaningful statement in terms of energy density of the Si/C composites, it is important to consider their volume in the lithiated state [10,66]. This is important as Si expands severely when it alloys with lithium. In this regard, we assume that the synthesized Si/C composites benefit from the fact that the Si is incorporated in carbon and, thus, these composites are expected to show quite small volumetric changes compared to composites where the Si is not embedded in the carbon.

### Electrochemical investigations of Si/C vs lithium metal

**Figure 4:**
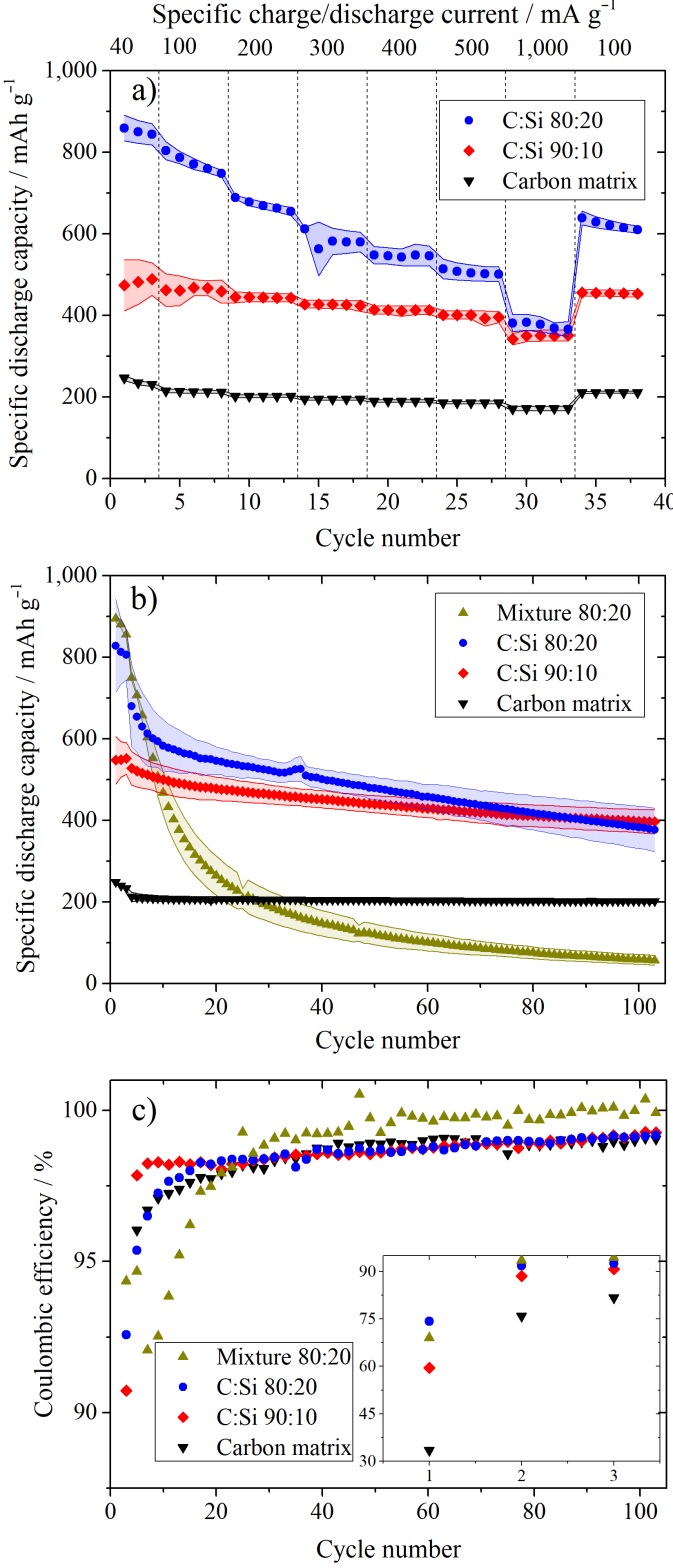
Constant current rate performance investigations at different charge/discharge currents (a) of the Si/C composites with a carbon to silicon ratio of 100:0, 90:10, 80:20 and constant current long-term cycling experiments (b) at a specific charge/discharge current of 400 mA g^−1^ after three formation cycles at 80 mA g^−1^ and the corresponding Coulombic efficiencies (c). In addition to the synthesized materials, a physical mixture of the pure carbon matrix and the Si-NPs is shown in b and c. CE and RE: metallic lithium; potential range 0.02 V and 1.5 V vs Li/Li^+^ (a) and 0.01 V and 1.5 V vs Li/Li^+^ (b, c).

The Li-ion storage capabilities of the different materials were investigated in symmetrical rate performance experiments (Figure 4a) with specific charge (=lithiation)/discharge (=delithiation) currents between 40 mA g^−1^ and 1,000 mA g^−1^ and in constant current long-term cycling investigations (Figure 4b). 100 charge/discharge cycles at a specific current of 400 mA g^−1^ were performed after three formations cycles with a formation current of 80 mA g^−1^. In Figure 4a, the excellent rate performance of the pure amorphous carbon matrix can be seen with only a low capacity decrease at high charge/discharge rates. At a current of 100 mA g^−1^, a capacity of ≈215 mAh g^−1^ is reached that is only slightly reduced to ≈172 mAh g^−1^ at the highest charge/discharge rate of 1,000 mA g^−1^, which corresponds to a C-rate of 4.65C considering a practical capacity of 215 mAh g^−1^. Through the addition of Si, a significant increase in capacity is achieved with capacities of ≈470 mAh g^−1^ and ≈770 mAh g^−1^ at a specific current of 100 mA g^−1^ for the C:Si 90:10 and C:Si 80:20 composite, respectively. These capacities are in a comparable range to the specific capacities achieved by Cakan et al. of ≈160 mAh g^−1^ for a pure hydrothermal carbon and 460 mAh g^−1^ for a Si/C composite with a Si content of ≈15 wt % at a specific current of 300 mA g^−1^ for 20 cycles [41].

The capacity decrease of the Si/C composites with increasing current rate is stronger compared to the pure carbon matrix, especially for the C:Si 80:20 composite, and therefore, can be directly related to the Si content of the samples. The CEs, voltage efficiencies (VEs) and energy efficiencies (EEs) of the pure carbon matrix, the C:Si 90:10, and C:Si 80:20 composite in the rate performance experiments are summarized in Figure S2 (Supporting Information File 1). The EEs and VEs were calculated as described by Meister et al. [8], using a virtual lithium iron phosphate (LFP) cathode with a potential of 3.4 V vs Li/Li^+^ as the positive electrode. From Figure S2, it can be seen that the pure carbon matrix (Figure S2a) exhibits the highest VE at each specific charge/discharge current, while the VE slightly decreases with the Si content, meaning that the C:Si 80:20 (Figure S2c) composite shows the lowest VE at all specific currents. At the highest specific charge/discharge rate of 1,000 mA g^−1^ all materials show the lowest VE with ≈96% for the pure carbon matrix (Figure S2a), ≈92% for the C:Si 90:10 (Figure S2b) and ≈90% for the C:Si 80:20 composite (Figure S2c). A similar trend can be observed regarding the correlation between the Si content and the CE, with the pure carbon matrix showing the highest CE and the C:Si 80:20 composite showing the lowest CEs at different specific charge/discharge currents, except for the formation cycles. Because of the higher VE and CE, the pure carbon matrix also shows the highest EE at different specific charge/discharge currents after the formation cycles.

In Figure 4b, the pure carbon matrix reveals a stable capacity of ≈200 mA g^−1^ with only minor capacity decay during the long-term cycling at a charge/discharge current of 400 mA g^−1^ and a capacity retention of ≈95% after the 103rd cycle referred to the 4th cycle (first cycle after formation). The Si/C composites, however, suffer from a stronger capacity decay that is again more pronounced with higher Si content, leading to a capacity retention of ≈75% and ≈55% for the C:Si 90:10 and C:Si 80:20 composite. A slightly higher capacity retention of ≈77% after 100 cycles was reported by Shen et al. [37] at a specific current of 200 mA g^−1^ for a pomegranate-inspired Si/C composite with a porous hydrothermal carbon matrix and a Si content of ≈10 wt %, retaining a capacity of 581 mAh g^−1^. For a reasonable comparison regarding the capacity retention of different materials, it should be considered though that in our experiments higher currents of 400 mA g^−1^ were applied during the long-term cycling experiments and electrodes with higher active material content of 90 wt % were used.

The slightly higher capacities of the different materials in the long-term performance investigations compared to the rate performance experiments can be explained with a different lower cut-off potential of 0.01 V vs Li/Li^+^ compared to 0.02 V vs Li/Li^+^ in the rate performance studies, which was chosen to avoid Li-metal plating at high charging rates. In order to verify if the incorporation of the Si into the carbon has a beneficial effect, a physical mixture of the pure carbon matrix and the pure Si-NPs was prepared in a ratio of 80:20, where the Si-NPs did not take part in the hydrothermal process. This mixture shows the highest capacity of all investigated materials with ≈750 mAh g^−1^ in the fourth cycle, but suffers at the same time from by far the strongest capacity decay (see Table 1). After 12 cycles the capacity already drops below the capacity of the C:Si 90:10 composite and after 35 cycles the capacity is even lower than that of the pure carbon matrix. The capacity retention after the 103rd cycle amounts to only ≈8% referring to the capacity in the 4th cycle. These results point out the highly beneficial effect of embedding Si into a carbon matrix. It has to be noted that the physical mixture as a “non-optimized system” might not be the optimum “reference system”, however, it clearly shows the improvement of embedding Si into the carbon matrix. Overall, many different factors (specific surface area, particle size, porosity, Si content, mass loading, etc.) of the reference system should be comparable to the prepared materials for a fair comparison, thus, it is rather difficult to find any suitable reference material.

**Table 1:** Overview of the specific discharge (=delithiation) capacities, capacity retention and first cycle Coulombic efficiencies of the different samples during the long-term cycling investigations.

Active material	Specific discharge capacity / mAh g^−1^			Capacity retention / %	Coulombic efficiency / %
	1st cycle	4th cycle	103rd cycle	4th to 103rd cycle	1st cycle

carbon matrix	249 ± 1	213 ± 9	202 ± 5	95 ± 2	34 ± 3
C:Si 90:10	548 ± 47	528 ± 33	397 ± 24	75 ± 2	60 ± 6
C:Si 80:20	829 ± 90	680 ± 80	377 ± 43	55 ± 13	74 ± 4
mixture C + Si 80:20	895 ± 8	750 ± 5	57 ± 10	8 ± 1	69 ± 1

The CEs determined during the long-term cycling tests are summarized in Figure 4c. The first cycle CE is ≈34% for the pure carbon matrix, whereas it amounts to ≈60% and ≈74% for the C:Si 90:10 and C:Si 80:20 composites. Even lower initial CEs of ≈52% and ≈40% for comparable hydrothermal carbon based Si/C composites were reported by Shen et al. [37] and Cakan et al. [41] for composites with a Si-content of 10 wt % and 15 wt %, respectively. The low CEs values can be explained with the high surface areas of the materials due to the small particle sizes and the presence of functional groups that can irreversibly consume Li-ions [25]. The higher CEs with higher Si content can be correlated to the BET surface areas of 367 ± 7 m^2^ g^−1^ for the C:Si 80:20 sample and 402 ± 7 m^2^ g^−1^ for the pure carbon matrix and are also in agreement with the SEM images (Figure 1), indicating that the surface area decreases with the Si content, due to morphological changes of the carbon matrix.

### Morphological changes during lithiation/delithiation

**Figure 5:**
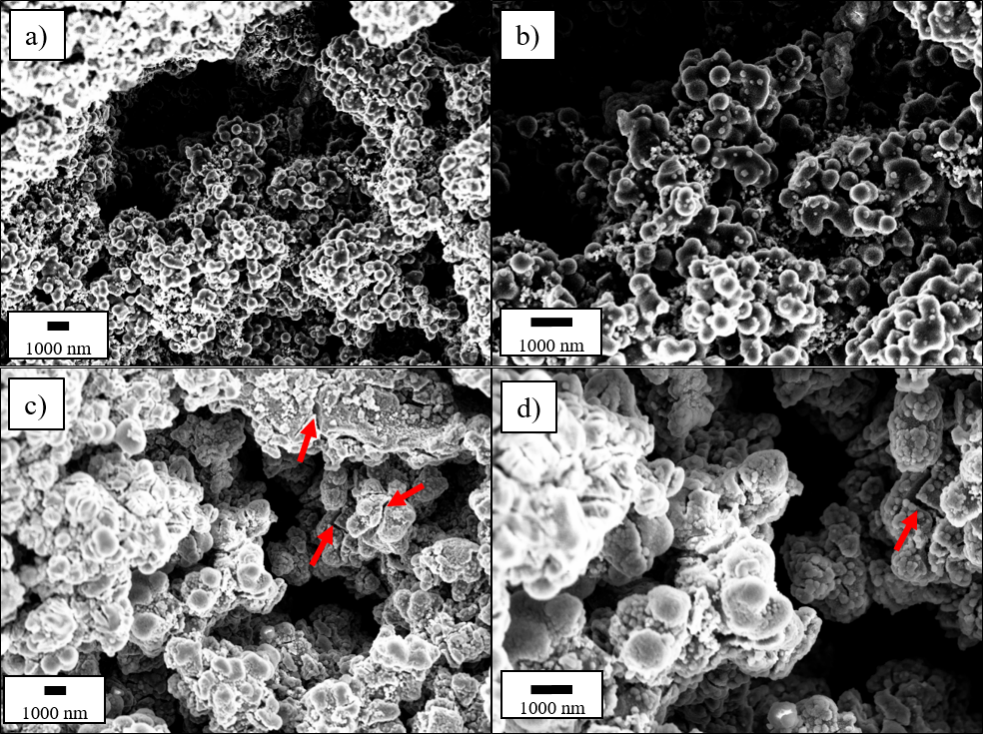
SEM micrographs of cycled electrodes after 13 cycles (including 3 formation cycles) of the C:Si 90:10 (a, b) and C:Si 80:20 composite (c,d).

With the aim to understand the reason for the strong capacity decay with increasing Si content, cycled electrodes were examined by means of SEM after 13 charge/discharge cycles (including three formation cycles), as presented in Figure 5. In Figure 5c and 5d, the electrode of the C:Si 80:20 composite exhibits several cracks (marked by red arrows in Figure 5c) that cannot be found on the surface of the electrode in Figure 5a and 5b, showing the C:Si 90:10 composite. It can be concluded that the mechanical stress caused by the volume expansion of the Si during the lithiation/delithiation process cannot be completely buffered by the amorphous carbon matrix and results in the formation of cracks when the Si content is too high, which is the case when the Si content is ≈20%. The crack formation during the lithiation/delithiation process is accompanied by severe consequences such as mechanical and electronic contact loss and pulverization of the active material, the trapping of Li inside detached Si, exposure of fresh Si to the electrolyte and breaking and reformation of the solid electrolyte interphase (SEI) layer [16,21–22,24]. All these factors contribute to an ongoing capacity loss with each cycle, leading to poor capacity retention. With higher Si content, the factors increase accordingly and explain the stronger fading of the C:Si 80:20 composite.

### Si/C vs NMC-111 full cell investigations

**Figure 6:**
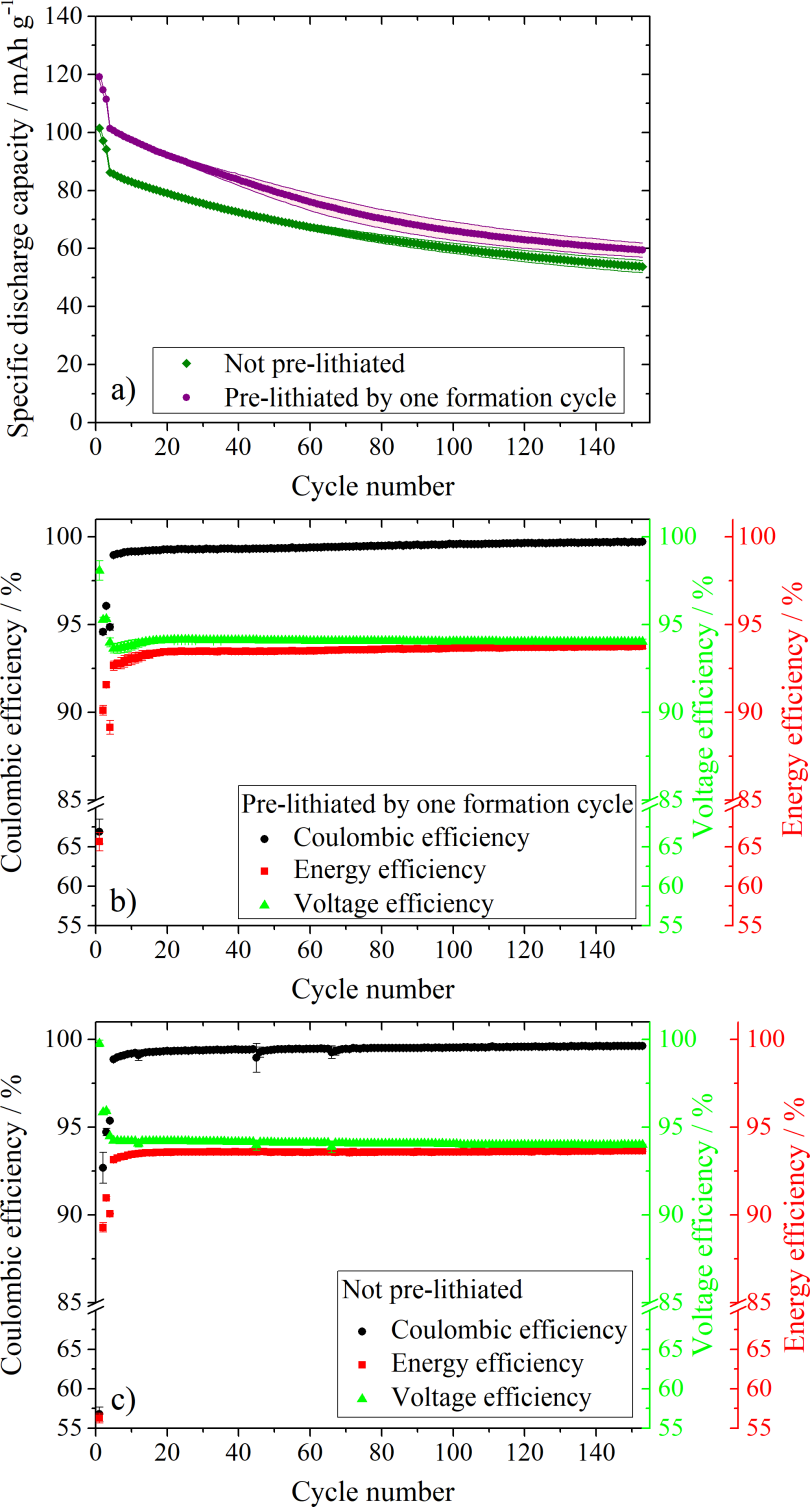
Constant current cycling of prelithiated (a, b) and non-prelithiated (a, c) C:Si 90:10 negative electrodes vs NMC-111 positive electrodes at a charge/discharge current of 100 mA g^−1^ after three formation cycles at 10 mA g^−1^. RE: metallic lithium, cut-off voltages: 3.0 V and 4.3 V; In b) and c) the VE and EE of the prelithiated (b) and non-prelithiated (c) full cells are presented.

The anode and cathode in a LIB full cell mutually affect each other [67]. LIB full cell investigations were carried out using positive electrodes containing NMC-111 as active material in combination with the C:Si 90:10 composite as the negative electrode. The C:Si 90:10 composite was chosen over the C:Si 80:20 composite due to the improved capacity retention in the previously shown investigations. In general, the anode/cathode capacity balancing of LIB full cells needs to be tailored in order to achieve the maximum energy density, but should avoid safety issues such as lithium-metal plating at the anode [68]. In our experiments, the negative electrode was not overbalanced in regard to the capacity of the positive electrode, since the first cycle CE of the C:Si 90:10 composite is quite low with ≈60%, as is known from the electrochemical investigations vs Li-metal (see Figure 4c). This leads to a relatively high consumption of active lithium from the cathode during the formation process [25] and, hence, a low risk of lithium metal plating at the anode. Additionally, the influence of electrochemical prelithiation on the cycling performance was investigated, as shown in Figure 6. Electrochemical prelithiation was, therefore, carried out via charging/discharging the C:Si 90:10 electrode vs Li-metal for one formation cycle, followed by the assembling of the full cell. In Figure 6a, the cycling performance of the prelithiated and non-prelithiated LIB full cells is compared, while Figure 6b and 6c summarize the corresponding CEs, VEs and EEs for the prelithiated (Figure 6b) and non-prelithiated (Figure 6c) full cells. In general, it can be stated that the discharge capacity of the prelithiated full cell is shifted to higher values. Despite the fact that the discharge capacity difference between prelithiated and non-prelithiated full cells diminishes with ongoing cycling, the discharge capacity of the prelithiated full cells is still higher than that of the non-prelithiated full cell even after 150 cycles. A similar trend of diminishing capacity differences between prelithiated and non-prelithiated full cells with ongoing cycling, can also be found in a publication by Kim et al., using a carbon-coated silicon monoxide anode vs a Li[Ni_0.8_Co_0.15_Al_0.05_]O_2_ cathode [69]. Even though prelithiation of Si-based anodes has become a huge research field in the recent years, there is still a lack of publications dealing with the effect of prelithiation on the long-term performance of LIB full cells using Si-based anodes. Thus, further investigations are necessary to identify the reasons for the stronger fading of the prelithiated full cells. In the context of optimizing the cycling performance of LIB full cells with Si-containing anode materials, it is important to take into consideration that prelithiation can not only be used to compensate the irreversible capacity loss in the first cycle, but also to generate a Li reservoir which can have a significant influence on the long-term performance of the cell [56].

In the first cycle, a discharge capacity of 101 mAh g^−1^ is achieved for the non-prelithiated full cell with a first cycle CE of 57%, while the prelithiated full cell delivers a discharge capacity of 119 mAh g^−1^ with a first cycle CE of 67%. The EE, VE and CE were determined in accordance to the procedure described by Meister et al. [8]. There it was shown that the EE can be calculated as the product of the CE and VE. The main differences between the prelithiated and non-prelithiated cell can be again found in the first cycle. The prelithiated full cells obtain an EE of ≈66%, while the EE of the non-prelithiated cells is just ≈56% in the first cycle, which is strongly influenced by differences of the CE. However, in general it can be stated that the development of the EE, VE and CE in dependence of the cycle number, are very similar for the prelithiated and non-prelithiated full cells. In the first cycle the VE reaches the highest value, higher than 98%, and then slightly decreases to ≈95% for the next two formation cycles. After the formation cycles, the VE is lower than before and stabilizes at ≈94% in both cases. The decrease compared to the formation cycles is most likely due to stronger polarization effects of the electrodes at higher currents [8,70]. A high VE indicates a small voltage hysteresis between charge and discharge of the cell. After the formation, the EE and VE are very similar to each other, with the VE being slightly higher in each cycle. The EEs and VEs obtained in this work are in a very similar range to those reported by Meister et al. for hard carbon and graphite anodes, where a virtual lithium iron phosphate cathode was used as the positive electrode for calculation [8].

**Figure 7:**
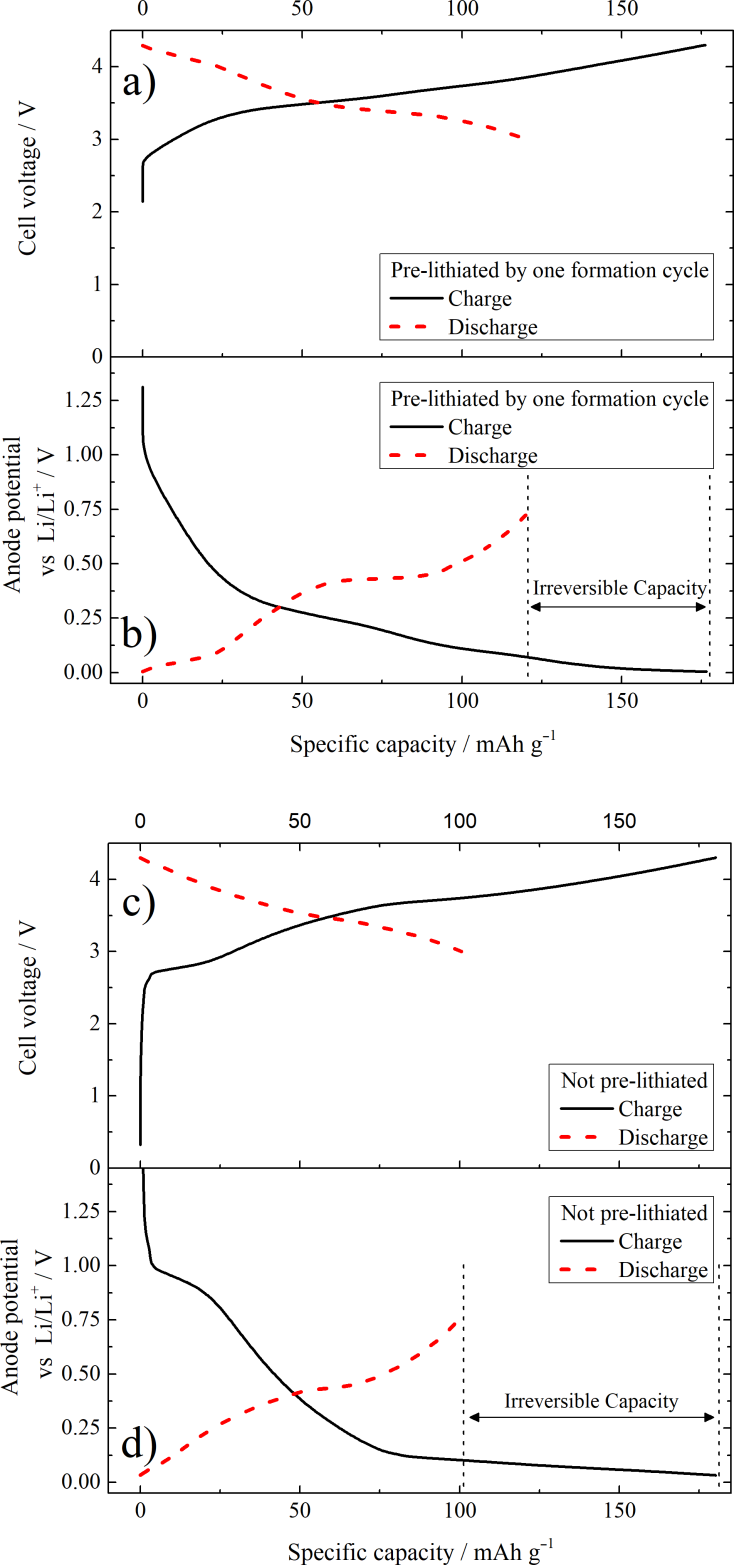
First cycle cell voltage (a, c) and anodic potential (b, d) profile using a full cell set-up with a prelithiated (a, b) and a non-prelithiated (c, d) C:Si 90:10 composite negative electrode (anode) vs a NMC-111 positive electrode (cathode). RE: metallic lithium, cut-off voltages: 3.0 V and 4.3 V.

Figure 7 presents the cell voltage, as well as the anode potential of the prelithiated (Figure 7a and 7b) and non-prelithiated (Figure 7c and 7d) LIB full cells as a function of the specific capacity during the first charge/discharge process. The main difference between the prelithiated and non-prelithiated full cells is the presence of a sloping plateau at ≈1 V vs Li/Li^+^ in the anode potential profile of the non-prelithiated full cell (Figure 7d). This can be attributed to the formation of the SEI by electrolyte decomposition. The absence of this plateau in Figure 7b is due to the fact that a major part the SEI is already formed during the prelithiation step before the first electrochemical charge. The presence/absence of the same plateau is also reflected in the cell voltage vs the specific capacity plot in Figure 7a and 7c at ≈2.7 V. The prolonged discharge plateau at ≈0.45 V vs Li/Li^+^ in the anode potential profile for the prelithiated full cell that originates from the delithiation of lithiated silicon (transition from crystalline to amorphous Si) depicts another difference between the prelithiated and non-prelithiated full cell.

**Figure 8:**
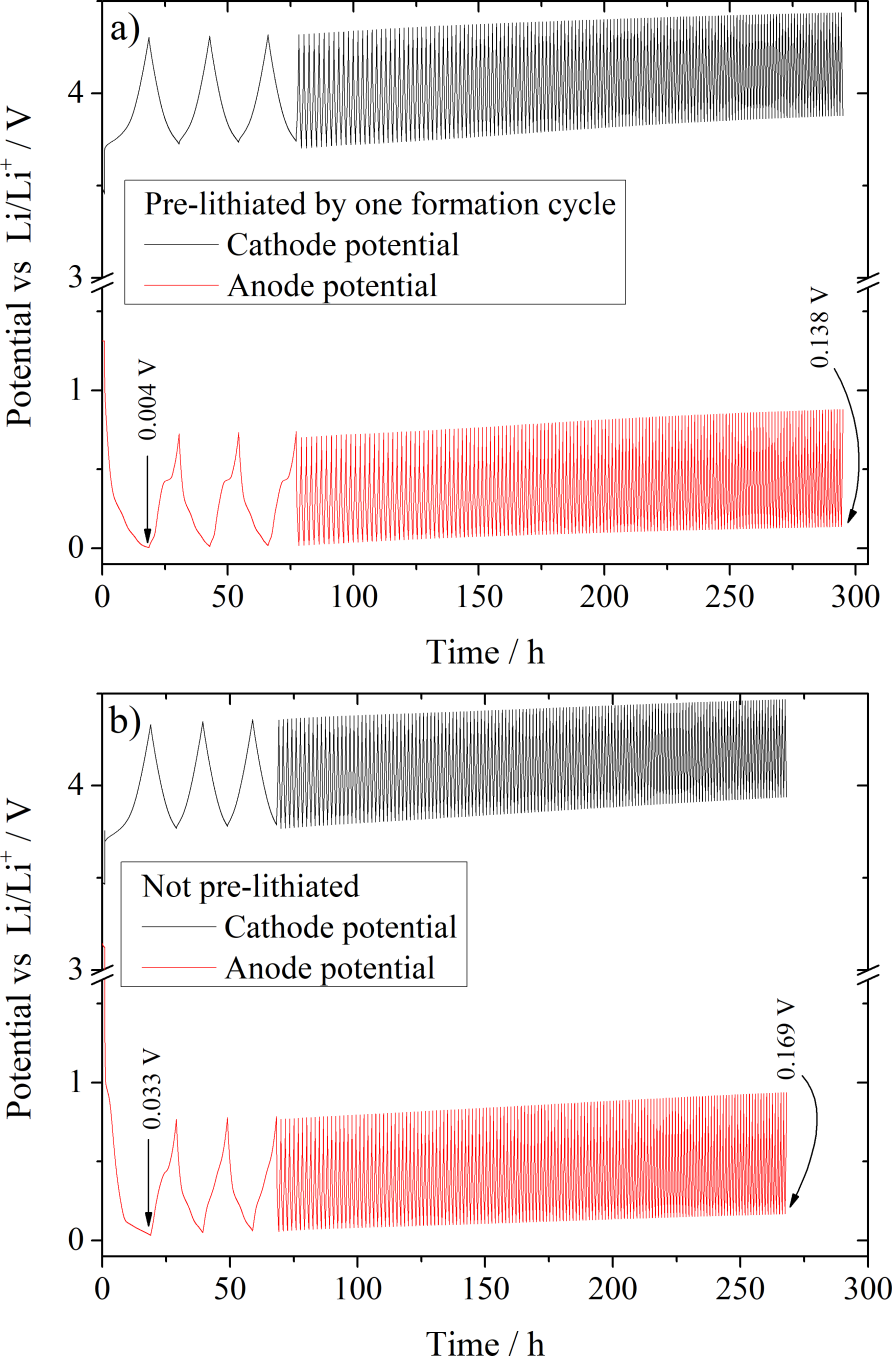
Development of the anode (negative electrode) and cathode (positive electrode) potential vs Li/Li^+^ in dependence of the time during cycling using a LIB full cell set-up with a prelithiated (a) and a non-prelithiated (b) C:Si 90:10 composite anode vs a NMC-111 cathode. RE: metallic lithium, cut-off voltages: 3.0 V and 4.3 V.

Figure 8 presents the development of the anode and cathode potentials of the prelithiated (a) and non-prelithiated (b) full cells vs time during cycling. With ongoing cycling, a shift of the end of charge (EOC) potential to higher potentials occurs in both cases. For the prelithiated full cell, the EOC anode potential in the first cycle is 0.004 ± 0.002 V vs Li/Li^+^, while the anode potential in the non-prelithiated full cell reaches a EOC potential of just 0.033 ± 0.002 V vs Li/Li^+^. This is a direct consequence of the lower amount of available active lithium in the non-prelithiated system, as more of the active Li is consumed during the SEI formation (lower first cycle CE) and, therefore, cannot be stored in the anode [25]. After 153 cycles, the EOC anode potential is considerably higher than that in the first cycle and reaches potentials of 0.138 ± 0.004 V vs Li/Li^+^ and 0.169 ± 0.004 V vs Li/Li^+^ for the prelithiated and non-prelithiated system, respectively. This results in a very comparable potential increase of 0.134 V and 0.136 V with respect to the first cycle for the prelithiated and non-prelithiated system. The EOC potential shift towards higher values most likely arises from a continuous loss of active lithium with the consequence that the anode gets less and less lithiated with ongoing cycle number. Therefore, the lithiation already stops at a higher potential than in the cycle before. As a consequence of the anode potential shift, the cathode potential is likewise shifted to higher potentials because of the constant cell voltage range of 3.0 V and 4.3 V during cycling. The detrimental consequences of such a voltage shift has been described in detail by Krüger et al. [67] and later by Beattie et al. [71].

Even though the first cycle CE is still quite low at ≈67% after the prelithiation, and thus requires further optimization, the high importance of this method regarding the application of Si-based anode materials with low initial CE is clearly discernable.

## Conclusion

In this study, we investigated a facile synthesis approach where Si-NPs are embedded into an amorphous carbon matrix via a hydrothermal process. The aim of the applied synthesis route was to obtain Si/C composite materials that combine the advantageous properties of Si and C. In summary, it can be stated that a strong improvement in capacity retention could be achieved compared to a mixture of Si and carbon in which Si-NPs were not incorporated into the matrix. At the same time though, the capacity fading was still observed with ongoing cycling. Especially the sample with the highest Si content of ≈20 wt % suffered from quite strong capacity decay due to the inability of the carbon matrix to buffer the volume changes of the Si-NPs sufficiently. This resulted in mechanical stress and the formation of cracks within the electrodes, as well as continuous SEI formation. Despite the fact that the initial Coulombic efficiency of the synthesized materials was quite low, we could show that these materials are applicable as anode material in a LIB full cell set-up vs NMC-111 cathodes with limited lithium content. Further, the performance could be improved by prelithiation of the anode. Even though the reported results indicated that the presented approach is limited to the use of small amounts of Si (less than 20 wt %), one should consider that there are many potential modifications of the synthesis process that could affect the mechanical properties of the carbon matrix. By changing the temperature, holding time, heating rate or stirring rate, the morphology and particle size of the formed carbon can be adjusted, which might lead to a more flexible, porous and stable matrix. However, further systematic investigations are necessary to identify the influence of different reaction parameters on the structure and morphology of the formed carbon matrix in order to optimize the electrochemical performance of hydrothermal-derived Si/C composites.

## Supporting Information

Additional figure, showing SEM micrographs of the physical mixture of Si and C in different magnifications, and a table, summarizing the tap densities of the C:Si composites. Additional figure showing the CEs, EEs and VEs in the charge/discharge current experiments for the Si/C composites with a carbon to silicon ratio of 100:0 (a), 90:10 (b) and 80:20 (c).

File 1 Supporting information.
