# Supplementary material for: Hydrothermal-derived carbon as a stabilizing matrix for improved cycling performance of silicon-based anodes for lithium-ion full cells
Source: Beilstein J Nanotechnol. 2018 Sep 5;9:2381–95. doi: 10.3762/bjnano.9.223 (PMC6142743; doi:10.3762/bjnano.9.223)
Supplement: File 1 — Supporting information. [file Beilstein_J_Nanotechnol-09-2381-s001.pdf]

Supporting Information
for

Hydrothermal-derived carbon as a stabilizing matrix for improved cycling performance of silicon-based anodes for lithium-ion full cells

Mirco Ruttert[1], Florian Holtstiege[1], Jessica Hüsker[1], Markus Börner[1], Martin Winter*[1,2,§] and Tobias Placke*[1,¶]

Address: [1]University of Münster, MEET Battery Research Center, Institute of Physical Chemistry, Corrensstraße 46, 48149 Münster, Germany and [2]Helmholtz Institute Münster, IEK-12, Forschungszentrum Jülich GmbH, Corrensstraße 46, 48149 Münster, Germany

Email: Tobias Placke - tobias.placke@uni-muenster.de; Martin Winter - martin.winter@uni-muenster.de
*Corresponding author

[§]m.winter@fz-juelich.de, Tel.: +49 251 83-36031, Fax: +49 251 83-36032

[¶]Tel.: +49 251 83-36826, Fax: +49 251 83-36032

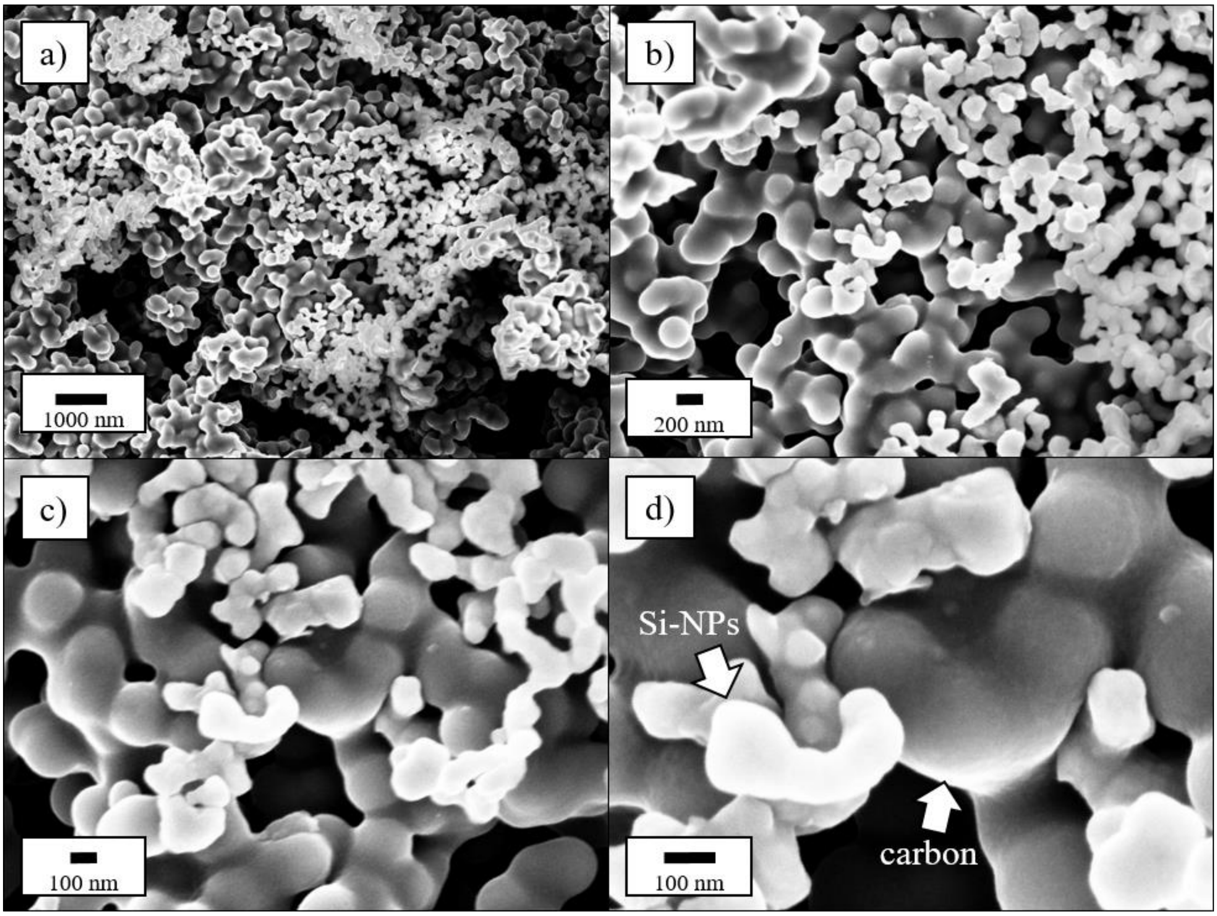

Figure S1: SEM micrographs of a physical mixture of the pure carbon matrix and the pure Si-NPs in a weight ratio of 80:20 in a magnification of 10k× (a), 25k× (b), 50k× (c) and 100k× (d).

Table S1: Overview of the tap densities of different materials used in this work.

Sample	Tap density / g cm^{-3}
Pure carbon matrix	0.16 ± 0.01
C:Si 90:10	0.19 ± 0.01
C:Si 80:20	0.24 ± 0.01
Physical mixture 80:20	0.20 ± 0.01
Pure Si-NPs	0.13 ± 0.01

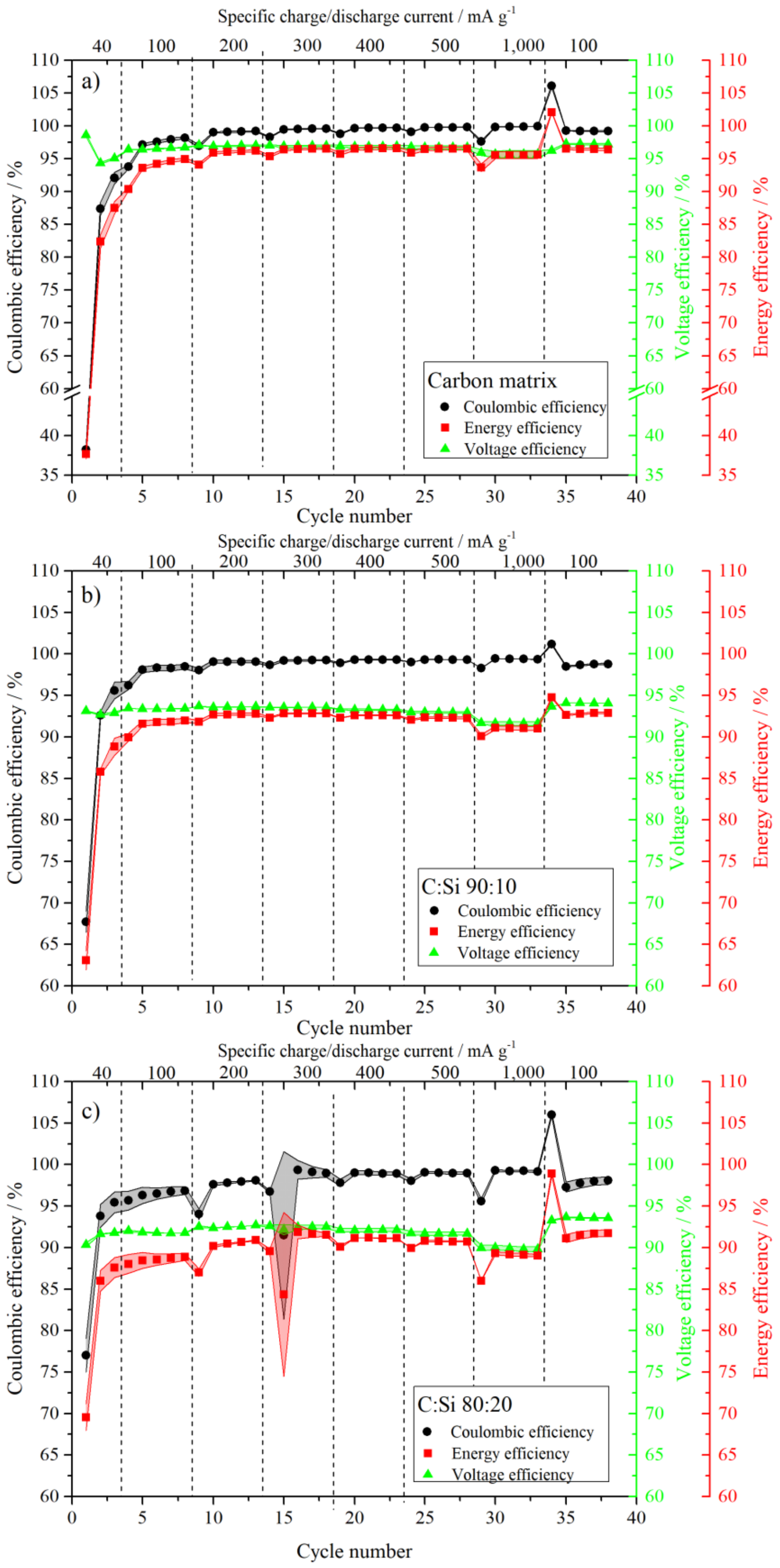

Figure S2: CEs, VEs and EEs of the Si/C composites with a carbon to silicon ratio of 100:0 (a), 90:10 (b), 80:20 (c) in constant current rate performance investigations at different charge/discharge currents (a). CE and RE: metallic lithium; potential range 0.02 V and 1.5 V vs Li/Li^+.
